# Pediatric Moyamoya Disease in Nepal and Challenges in a Resource‐Limited Setting: A Case Report

**DOI:** 10.1002/ccr3.70947

**Published:** 2025-10-06

**Authors:** Kapil Khanal, Sunil Dhungana, Bindu Gyawali, Rishab Khanal, Binit Bajracharya, Nishant Neupane, Nijan Sharma, Kailash Mani Pokhrel, Manish Acharya, Shreya Sinha

**Affiliations:** ^1^ Maharajgunj Medical Campus (MMC), Institute of Medicine (IOM) Tribhuvan University (TU) Kathmandu Nepal; ^2^ Department of Pediatric Medicine Maharajgunj Medical Campus (MMC), Institute of Medicine (IOM), Tribhuvan University (TU) Kathmandu Nepal; ^3^ Department of Emergency Medicine Maharajgunj Medical Campus (MMC), Institute of Medicine (IOM), Tribhuvan University (TU) Kathmandu Nepal

**Keywords:** antiplatelet therapy, cerebrovascular disorder, conservative management, moyamoya disease, pediatric stroke, transient ischemic attack

## Abstract

Moyamoya disease (MMD) is a rare progressive cerebrovascular disorder characterized by stenosis of the terminal internal carotid arteries with fragile collateral vessel formation. It is common in East Asia but underreported in Nepal. Pediatric cases typically present with ischemic symptoms such as transient ischemic attacks, stroke, or seizures. We report a 7‐year‐old Nepali boy with 11 days of fever followed by recurrent tonic–clonic seizures, progressive right‐sided weakness, and impaired fine motor function. Examination showed right hemiparesis with an upgoing plantar reflex. MRI and MR angiography revealed multiple acute infarcts with supraclinoid internal carotid artery stenosis and extensive collateral vessels, confirming MMD. Cerebrospinal fluid and autoimmune workup were unremarkable. The child was managed conservatively with aspirin and levetiracetam, leading to partial recovery. Surgery was deferred due to financial limitations and initial improvement. This case underscores the importance of considering MMD in pediatric strokes in non‐endemic regions. In resource‐limited settings like Nepal, conservative treatment offers symptomatic relief, but long‐term follow‐up is essential to monitor progression and surgical needs.

**Summary**

- Early recognition of Moyamoya disease in pediatric stroke is essential, even in non‐endemic regions like Nepal.
- While surgical revascularization is the definitive treatment, conservative management may provide short‐term improvement.
- However, long‐term follow‐up is vital due to the disease's progressive nature and risk of future neurological deterioration.

## 1 Introduction

Moyamoya disease (MMD) is a chronic, progressive cerebrovascular disorder. It is characterized by narrowing of the terminal intracranial portion of the internal carotid artery and the circle of Willis, leading to the formation of fragile collateral vessels [1]. The term “Moyamoya,” introduced by Suzuki and Takaku in 1969, means “something hazy like a puff of cigarette smoke drifting in the air,” in Japanese, and it refers to the hazy characteristic appearance of these collateral vessels on angiographic [2].

MMD is relatively common in people living in East Asian countries such as Korea and Japan, compared to those in the Western Hemisphere [3]. The disease has been reported in 1063 cases worldwide, excluding Japan. Among the 625 cases in Asia, 245 occurred in China and 289 in Korea, highlighting the disease's prominence in these countries [2]. However, only a few cases have been reported from countries like Nepal and India. The age of onset of the symptomatic disease has two peak distributions with different clinical presentations at 5 to 9 years of age and 45 to 49 years of age [1, 3]. The peak appears to occur later in women than in men [4].

In children, ischemic symptoms, especially transient ischemic attacks (TIAs), are predominant [5, 6]. Intellectual decline, seizures, and involuntary movements are also more common in this age group. In contrast, adult patients present with intracranial hemorrhage more often than pediatric patients [7]. TIAs are the most important clinical manifestation in both children and adults.

Magnetic resonance (MR) angiography and computed tomographic angiography are noninvasive diagnostic methods. High‐resolution vessel wall MR imaging also helps diagnose MMD by revealing concentric vessel wall narrowing with basal collaterals [3].

Treatments can be classified as conservative or interventional. For conservative management, treatment with antiplatelet drugs, anticonvulsant drugs, pain management, and rigid control of additional risk factors, such as dyslipidemia, hypertension, and diabetes, is highly recommended [8, 9]. For surgical treatment, revascularization procedures by direct or indirect methods can be adopted. In direct methods, the vessels (distal branches of ECA and distal branches of ICA) are typically temporarily clamped, an incision is made in both vessels, and anastomoses are done. While indirect techniques are dependent on the physiologic process of angiogenesis as this complex biochemical process occurs in response to wound healing and allows connections to form between adjacent damaged vessels [10, 11].

In this study, we present a case of a 7‐year‐old Nepali child who presented with fever, followed by tonic‐clonic seizure, right‐sided weakness impairing his ability to walk and perform fine motor tasks. He was later diagnosed as having MMD and was managed conservatively. The case is unique in the context of Nepal, as the disease is rare in this part of Asia. This case has been reported in line with SCARE 2023 criteria [12].

## 2 Case Presentation

A 7‐year‐old male child, previously in his usual state of health, presented with an 11‐day history of fever that was insidious in onset and progressively increased in severity, with a maximum recorded temperature of 101°F. The fever was not associated with chills, rigor, or diurnal variation. On the second day of illness, he developed abnormal body movements in the form of tonic–clonic seizures involving the right upper and lower limbs. These episodes lasted for 3 to 4 min and recurred multiple times but were not associated with loss of consciousness, generalization, or bowel and bladder incontinence. Following the seizure episodes, he developed right‐sided weakness, impairing his ability to walk and perform fine motor tasks. He was taken to a nearby hospital, where he was diagnosed with ischemic stroke and was started on aspirin and levetiracetam. His weakness partially improved with treatment, enabling him to run and move his limbs, but he remained unable to hold a glass properly and exhibited slurred speech. The patient was discharged from the hospital.

The day after discharge, his condition deteriorated with increased sleepiness during both day and night, a high‐grade fever reaching 103°F that did not respond to medications, and worsening right‐sided weakness, rendering him unable to walk or move his limb. There were no associated abnormal body movements, behavioral changes, bowel or bladder incontinence, difficulty chewing, hearing problems, feeding difficulties, shortness of breath, palpitations, visual disturbances, petechiae, purpura, rashes, or abnormal bleeding. Due to worsening symptoms, he was readmitted to a nearby hospital, but with no significant improvement, he was referred to our center for further management.

The past medical history was insignificant. He was born at term via cesarean section without complications during pregnancy or delivery, required no neonatal resuscitation or admission, and had no parental concerns regarding his developmental milestones. He was fully immunized as per the national protocol, including the COVID‐19 and typhoid conjugate vaccine (TCV). There was no family history of hypertension, stroke, diabetes mellitus, or similar neurological conditions. The child belonged to a family of eight members with no significant environmental exposures.

On general examination, he was in fair condition, with multiple small petechiae on the abdomen and mild pallor. His vital signs included a temperature of 98.6°F, a pulse of 102 beats per minute, a respiratory rate of 26 breaths per minute, and a blood pressure of 100/60 mmHg. Capillary refill time was normal. His anthropometric measurements showed a weight of 20 kg and a height of 115 cm, both within the normal range for age. On neurological examination, he was alert and oriented with a Glasgow Coma Scale (GCS) score of 15/15. His memory, language, and speech were intact. Muscle tone was decreased in the right upper and lower limbs, and muscle power was reduced to 3/5 on the right side while remaining normal on the left. His right plantar reflex was upgoing. Sensory examination was intact, and there were no signs of meningeal irritation.

## 3 Methodology (Investigations and Management)

**FIGURE 1:**
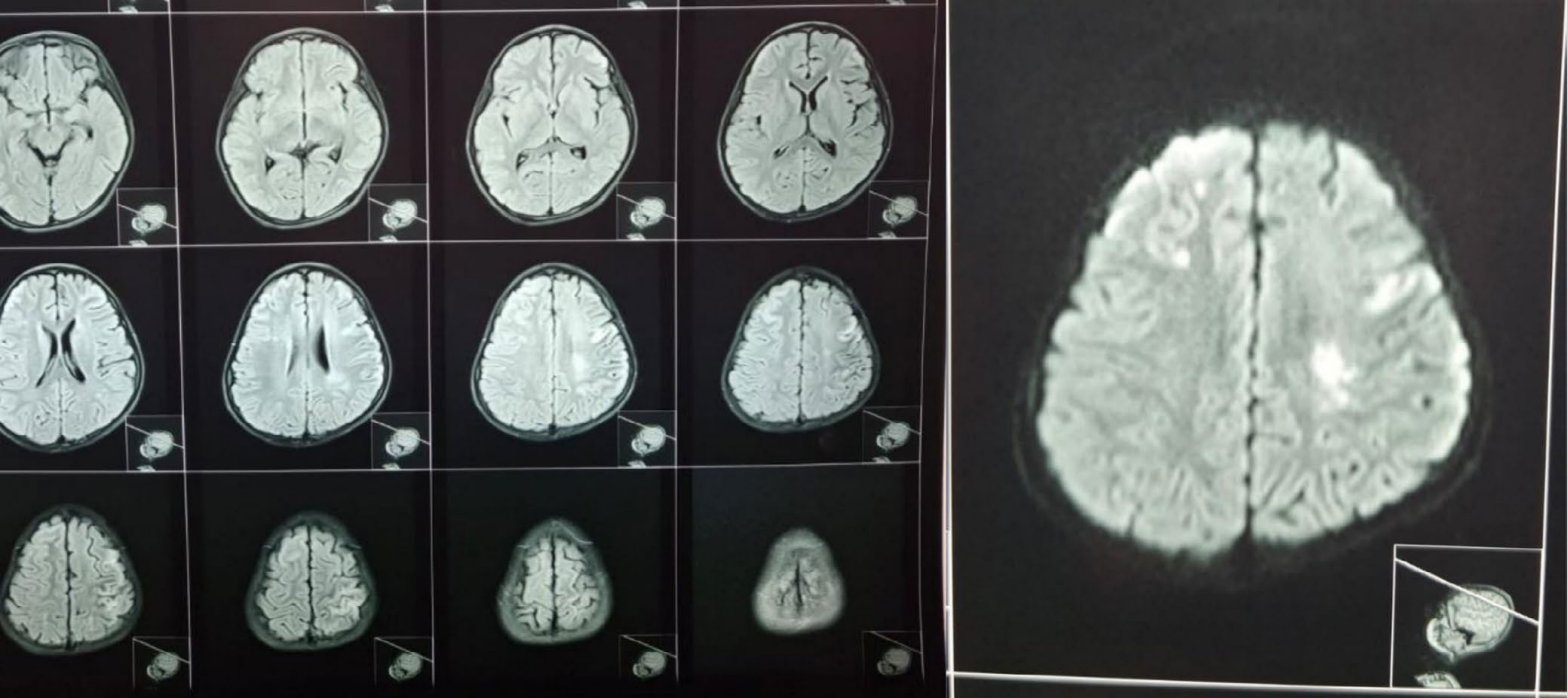
MRI multiple acute infarcts in the bilateral frontal and left parietal lobes.

**FIGURE 2:**
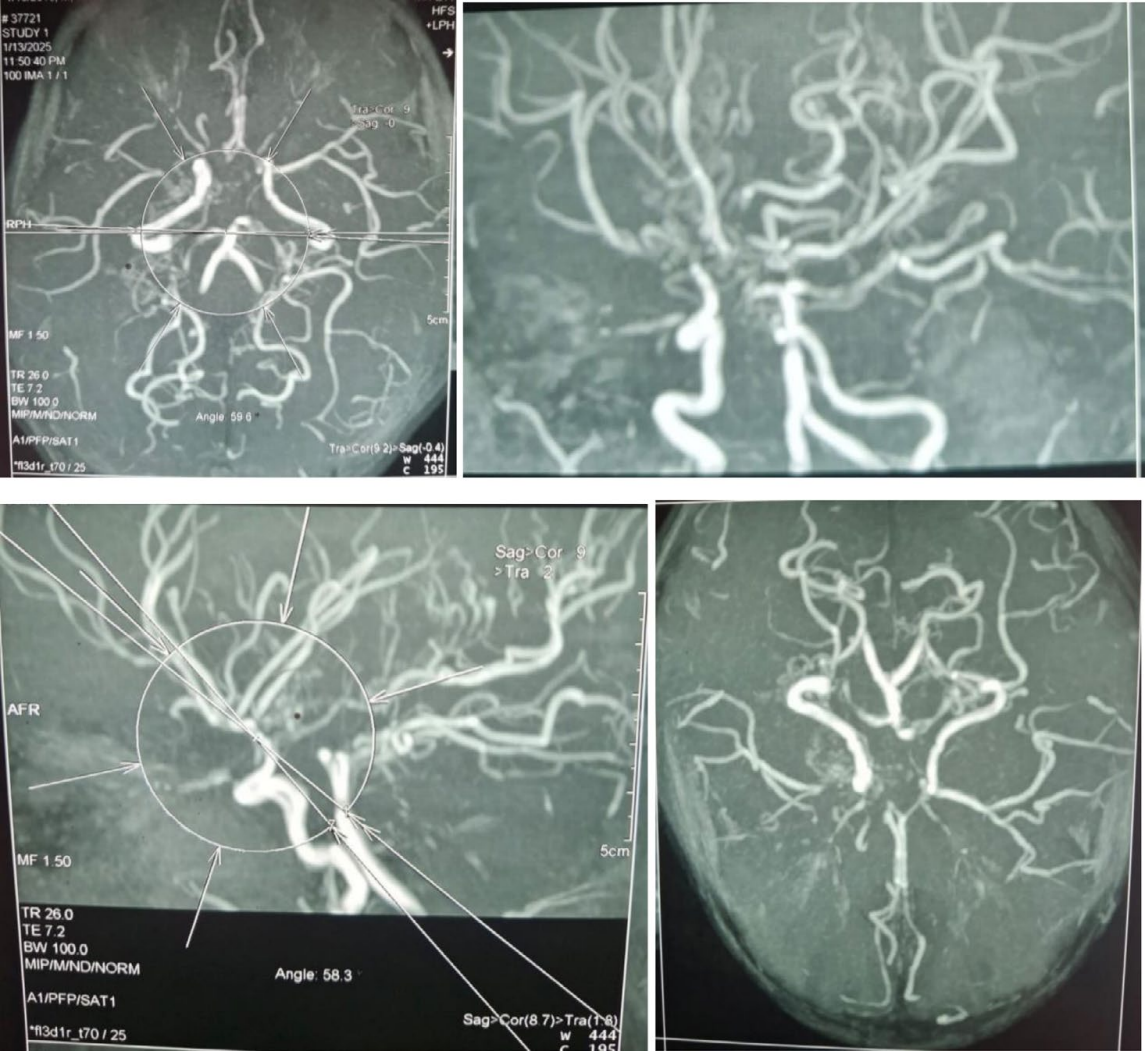
MR angiography showing an attenuated caliber with tapering of the supraclinoid and communicating segments of the bilateral carotid arteries (left more than right), an attenuated caliber of the A1 segment of the right anterior cerebral artery, and the M1 segment of the right middle cerebral artery. Multiple collaterals are present in the region of the distal internal carotid artery and right middle cerebral artery, consistent with the characteristic features of Moyamoya disease.

The child underwent baseline investigations, MRI (Figure 1), and MR angiography (Figure 2), which revealed multiple acute infarcts in the bilateral frontal and left parietal lobes. Imaging also demonstrated an attenuated caliber with tapering of the supraclinoid and communicating segments of the bilateral carotid arteries (left more than right), an attenuated caliber of the A1 segment of the right anterior cerebral artery, and the M1 segment of the right middle cerebral artery. Multiple collaterals were present in the region of the distal internal carotid artery and right middle cerebral artery, consistent with the characteristic features of MMD. Other investigations were unremarkable.

The child was admitted for observation and managed conservatively. He was started on antiplatelet therapy (aspirin) to prevent further ischemic events and anticonvulsants (levetiracetam) to control seizures. Supportive care was provided to optimize neurological recovery. Over the course of treatment, his symptoms improved, and he regained the ability to walk. However, residual weakness and slurring of speech were still present. Neurosurgical consultation was obtained, and given the child's clinical improvement, he was discharged with advice to continue his medications and to follow up closely for monitoring of disease progression and potential future interventions.

## 4 Discussion

MMD is a progressive cerebrovascular disorder characterized by stenosis of the terminal internal carotid arteries and the formation of fragile collateral vessels, predisposing patients to ischemic and hemorrhagic strokes [13, 14]. Although commonly reported in East Asian populations, MMD is a rare entity in Nepal, with limited case reports in the literature. This case of a 7‐year‐old Nepali child diagnosed with MMD underscores the importance of early recognition, appropriate diagnostic evaluation, and proper management strategies, particularly in resource‐limited settings.

Despite many advances, the exact pathophysiological triggers and precise timeframe of the progression of MMD remain unknown, although lines of evidence have indicated pathogenic pathways as diverse as those of angiogenesis, genetics, the immune system, and inflammation. MMD has been associated with several angiogenesis‐related factors, such as endothelial colony‐forming cells and cytokines, including vascular endothelial growth factor, transforming growth factor beta 1, basic fibroblast growth factor, and hepatocyte growth factor [8, 15]. The disease has been implicated with some genetic predispositions, particularly in East Asian populations, where familial cases account for up to 15% of all reported cases. The RNF213 gene mutation (p.R4810K variant) has been linked to MMD in Japanese, Chinese, and Korean populations [16].

The clinical symptoms of MMD in children were divided by Maki and Enomoto [17] into four categories: Infarction type, TIA, Bleeding type and Epileptic type, where Infarction and TIA types account for 70%–80% of cases. Our patient falls under both infarction and epileptic type according to this classification. Our patient falls under both infarction and epileptic type according to this classification. MMD has a bimodal age distribution at 5 to 9 years of age and 45 to 49 years of age [1]. Pediatric cases, as seen in our patient, primarily present with ischemic symptoms, including TIAs, strokes, and seizures [5, 6]. In contrast, adults are more likely to present with intracranial hemorrhages due to the rupture of fragile collateral vessels [7].

Our patient initially presented with fever, seizures, and right‐sided weakness, which progressed despite initial medical management. As per our knowledge, no literature exists at present that has probed the association between fever in MMD. However, Shoukat et al. have hypothesized that hyperthermia could induce vasoconstriction, which would have led to hypo‐perfusion in the patient, which in turn would trigger the symptoms in the patient [18, 19]. This is solely a hypothesis and would require further controlled experiments for confirmation. The combination of focal seizures and hemiparesis raised suspicion of a cerebrovascular event, but other differential diagnoses, including central nervous system (CNS) infections, autoimmune vasculopathies, and metabolic disorders, needed to be considered. CNS infections such as tuberculous meningitis, bacterial meningitis, and viral encephalitis are common in Nepal and can present with seizures, focal neurological deficits, and altered sensorium. However, the absence of meningeal signs, normal cerebrospinal fluid (CSF) analysis, and the presence of ischemic infarcts on imaging favored a vascular etiology.

Autoimmune conditions, such as primary angiitis of the central nervous system (PACNS), systemic lupus erythematosus (SLE), and antiphospholipid syndrome (APS), can also mimic MMD [20]. However, our patient had no history of recurrent thrombosis, autoimmune symptoms, or laboratory findings suggestive of an underlying inflammatory or autoimmune disorder.

Neuroimaging plays a pivotal role in the diagnosis of MMD. In our case, MRI and MR angiography revealed multifocal acute infarcts and characteristic vascular abnormalities, including stenosis of the supraclinoid and communicating segments of bilateral carotid artery and the presence of multiple collaterals, forming the classical “puff of smoke” appearance. This angiographic pattern and associated clinical features differentiate MMD from moyamoya syndrome, which refers to similar vascular changes secondary to underlying conditions such as Down syndrome, cranial irradiation, sickle cell disease, neurofibromatosis type 1, and thyroid disease, as well as less frequently, systemic lupus erythematosus, Turner syndrome, and Noonan syndrome [21]. The Suzuki staging system is commonly used to classify the severity of MMD based on angiographic findings [22]. In early stages, progressive narrowing of the internal carotid arteries leads to collateral vessel formation, whereas in later stages, major arteries may be completely occluded [23]. Our patient exhibited features consistent with moderate disease severity, with involvement of the anterior cerebral and middle cerebral arteries and extensive collateralization.

Currently, there is no drug treatment proven to delay, halt, or reverse the natural progression of MMD. Treatments can be classified as conservative or interventional tailored to the patient's clinical presentation and disease severity. For conservative management, treatment with antiplatelet drugs, anticonvulsant drugs, pain management, and rigid control of additional risk factors, such as dyslipidemia, hypertension, and diabetes, is highly recommended [8, 9]. The role of antiplatelets in pediatric MMD remains a subject of debate, as their efficacy in preventing stroke progression has not been firmly established. Kijpaisalratana et al. recently published a review that concluded that antiplatelet treatment in patients with MMD does not demonstrate a protective effect against ischemic type of MMD. Favorably, antiplatelet treatment does not increase the risk of hemorrhagic stroke in patients with MMD [24]. Considering this fact and the thromboembolic nature of ischemic strokes in MMD, antiplatelets are commonly prescribed as part of conservative management. In resource‐limited settings like Nepal, where surgical interventions may not be readily available, optimizing conservative treatment is crucial in preventing recurrent strokes and minimizing neurological deficits.

For surgical treatment, revascularization surgery is the mainstay of treatment for MMD, particularly in patients with recurrent strokes despite medical therapy. For revascularization, direct or indirect methods can be adopted. In direct methods, the vessels (distal branches of ECA and distal branches of ICA) are typically temporarily clamped, an incision is made in both vessels, and anastomoses are done. While indirect techniques are dependent on the physiologic process of angiogenesis as this complex biochemical process occurs in response to wound healing and allows connections to form between adjacent damaged vessels [10, 11]. In patients with MMD who experienced disease onset during childhood, extensive spontaneous collateral circulation often develops, increasing the risk of hemorrhagic stroke in later life [25]. The reduced regional cerebral blood flow (rCBF), especially in the left dorsolateral prefrontal cortex and left medial frontal cortex, has been significantly correlated with lower FIQ and Processing Speed Index (PSI). These findings show the importance of revascularization surgery in optimal neurocognitive development [25, 26, 27].

Although surgical intervention is preferred in symptomatic cases, its feasibility depends on the availability of specialized neurosurgical expertise and resources. In our case, neurosurgical consultation was sought, but given the child's initial improvement with medical therapy, a decision was made to continue conservative management with close follow‐up. Long‐term evaluation remains essential, as MMD is a progressive disease that may eventually require revascularization. Managing MMD in Nepal presents several challenges, including limited awareness among healthcare professionals, restricted access to advanced neuroimaging, and a lack of specialized neurosurgical centers. Many cases may go undiagnosed or misdiagnosed as other stroke etiologies, leading to delays in appropriate management. Establishing dedicated stroke care centers, improving access to MR angiography, and enhancing physician training on rare cerebrovascular diseases are essential steps in addressing these challenges. Financial constraints also pose a significant barrier to optimal care, as neurosurgical interventions are costly and may not be accessible to all patients. Government and nongovernmental initiatives aimed at subsidizing stroke care and expanding neurosurgical services in Nepal could improve outcomes for patients with MMD and other cerebrovascular disorders. Similarly, proper training in the field of surgical approach and a guideline incorporating the investigation and management of Moya Moya disease ensure standard surgical treatment for such cases.

In a similar case of pediatric MMD in Nepal where acute presentation with fever and confusion in an 8‐year‐old child was prescribed 75 mg aspirin once daily as a secondary prophylactic therapy for stroke and her fever was managed with antipyretics and adequate rest. As the patient improved, no revascularization surgery was performed [28]. In another ischemic case of Moyamoya in a 20‐year‐old from Nepal, the patient was initially given IV Normal Saline 3% along with IV Mannitol 200 mL to reduce the swelling in the brain as seen in MRI MRA and then aspirin 150 mg/day was prescribed. Weakness and facial deviation subsequently subsided [29].

This case report has several limitations. As a single case study, its findings cannot be generalized to a broader population, especially given the rarity of MMD in Nepal. Due to the patient's financial constraints, we were unable to investigate the specific causes or pathogenic pathways of MMD, such as abnormalities in angiogenesis, genetic factors, or immune system involvement. Additionally, the absence of long‐term follow‐up prevents a comprehensive assessment of disease progression and treatment outcomes. Moreover, while the report discusses early management, it does not provide insights into long‐term treatment strategies or patient prognosis.

## 5 Conclusion

MMD is a rare progressive cerebrovascular disorder that primarily affects the internal carotid arteries and their branches, leading to recurrent strokes, especially in children. Although the disease is most prevalent in East Asian countries, it is rarely reported in Nepal and neighboring regions. This case highlights the importance of recognizing MMD as a potential cause of pediatric stroke in Nepal. The patient's presentation with fever, seizures, and progressive hemiparesis underscores the need for early neuroimaging to establish an accurate diagnosis. Although medical management provided symptomatic relief, long‐term follow‐up remains crucial to evaluate disease progression and potential surgical intervention.

## Author Contributions

**Kapil Khanal:** data curation, investigation, methodology, project administration, supervision, visualization, writing – original draft, writing – review and editing. **Sunil Dhungana:** conceptualization, data curation, investigation, methodology. **Bindu Gyawali:** investigation, methodology, writing – original draft. **Rishab Khanal:** resources, supervision, writing – original draft. **Binit Bajracharya:** conceptualization, investigation, methodology, writing – review and editing. **Nishant Neupane:** methodology, writing – original draft. **Nijan Sharma:** methodology, writing – original draft. **Kailash Mani Pokhrel:** methodology, supervision, visualization, writing – original draft. **Manish Acharya:** investigation, writing – original draft. **Shreya Sinha:** investigation, visualization, writing – original draft.

## Disclosure

The authors have nothing to report.

## Consent

Written informed consent was obtained from the patient to publish this case report in accordance with the journal's patient consent policy.

## Conflicts of Interest

The authors declare no conflicts of interest.

## Data Availability

Data sharing is not applicable to this article, as no datasets were generated or analyzed during the current study.
